# Integrated fecal SDC2 methylation and serum tumor markers improve noninvasive detection of colorectal cancer

**DOI:** 10.3389/fonc.2026.1919347

**Published:** 2026-10-21

**Authors:** Shenglan Tong, Kang Rao, Man Sun, Yang Wu, Yan Dong, Changgang Xiang

**Affiliations:** Department of Laboratory Medicine, The First People’s Hospital of Jiangxia District, Wuhan City, Hubei University of Medicine, Wuhan, China

**Keywords:** colorectal cancer, early diagnosis, fecal SDC2 methylation, noninvasive screening, serum tumor marker

## Abstract

**Background:**

Fecal SDC2 methylation is a promising noninvasive biomarker for colorectal cancer (CRC), whereas serum tumor markers, including carcinoembryonic antigen (CEA), carbohydrate antigen 19-9 (CA19-9), CA242, and CA50, are primarily used for postoperative surveillance and have limited value as standalone early diagnostic markers. This study evaluated the diagnostic performance of combining fecal SDC2 methylation with these four serum markers for CRC detection.

**Methods:**

A total of 354 participants were enrolled, including 55 with CRC, 188 with adenoma/polyps, 106 with non-tumor/inflammatory conditions, and 5 with other undetermined lesions. Fecal SDC2 methylation was assessed by quantitative methylation-specific PCR, and serum markers were measured via electrochemiluminescence immunoassays. Diagnostic performance was evaluated using multivariable logistic regression and receiver operating characteristic (ROC) curve analysis. AUCs with 95% confidence intervals were calculated, and sensitivity, specificity, positive predictive value (PPV), and negative predictive value (NPV) were estimated at the Youden-optimal threshold.

**Results:**

Fecal SDC2 methylation levels were significantly higher in CRC patients compared to those with adenomas or non-tumor lesions. Fecal SDC2 Ct value alone yielded an AUC of 0.814 (95% CI, 0.751-0.876). The AUC increased to 0.866 (95% CI, 0.814-0.918) after addition of CEA and to 0.872 (95% CI, 0.818-0.926) for the full model incorporating CEA, CA19-9, CA242, and CA50. At the Youden-optimal threshold, the full model achieved a sensitivity of 87.3% (95% CI, 76.0%-93.7%), specificity of 78.6% (95% CI, 73.6%-82.9%), PPV of 42.9% (95% CI, 34.1%-52.1%), and NPV of 97.1% (95% CI, 94.2%-98.6%). In contrast, individual serum markers showed limited diagnostic performance, with AUC values of 0.709 (CEA), 0.666 (CA19-9), 0.495 (CA242), and 0.567 (CA50).

**Conclusion:**

Integration of fecal SDC2 Ct values with serum tumor markers showed a modest improvement in the noninvasive discrimination of CRC compared with SDC2 alone. Prospective external and stage-specific validation is required before clinical screening implementation.

## Introduction

Colorectal cancer (CRC) is the third most common malignancy and the second leading cause of cancer-related death globally, with nearly 1.9 million new cases and over 904,000 deaths annually (1). The vast majority of CRC cases arise from precursor lesions, namely colorectal adenomas, through a multistep accumulation of genetic and epigenetic alterations over a period of several years (2, 3). This protracted carcinogenic process provides a critical window of opportunity for early intervention. Early-stage CRC has a five-year survival rate exceeding 90%, whereas metastatic disease carries a dismal prognosis of approximately 15% (4). Therefore, early detection is crucial for reducing CRC mortality. Current screening methods include colonoscopy and fecal immunochemical testing (FIT). However, colonoscopy is invasive and resource-intensive, limiting compliance, while FIT shows suboptimal sensitivity for precancerous adenomas (5, 6). These limitations underscore the urgent need for non-invasive, accurate diagnostic tools.

In recent years, molecular biomarkers have emerged as promising non-invasive alternatives for cancer diagnostics. Among these, aberrant DNA methylation represents an early and frequent event in colorectal carcinogenesis, and its detection in stool offers several advantages, including tissue specificity, stability, and the ability to capture molecular alterations from exfoliated neoplastic cells (7). In the United States, the FDA-approved stool-DNA screening landscape is centered on multitarget stool DNA testing. Cologuard, the first FDA-approved multitarget stool DNA test, has been available since 2014 (8). In October 2024, the FDA approved Cologuard Plus™, a next-generation mt-sDNA test, for average-risk adults aged ≥45 years (9). The approval was supported by the prospective BLUE-C study involving more than 20,000 participants, in which Cologuard Plus achieved 93.9% sensitivity for CRC and 43.4% sensitivity for advanced precancerous lesions at 92.7% specificity (10). Other stool-based molecular assays, including ColoClear and SDC2-based tests such as EarlyTect, have been evaluated in different regulatory and clinical settings and provide complementary context for the evolving stool-DNA field (11, 12). Syndecan-2 (SDC2), a transmembrane heparan sulfate proteoglycan involved in cell proliferation and adhesion, has been identified as a sensitive and specific methylation biomarker for CRC (13). Methylation of the SDC2 promoter is prevalent in colorectal tumors, and fecal SDC2 methylation testing has demonstrated favorable diagnostic accuracy in prior studies (14). However, its performance may vary because of heterogeneous shedding of methylated DNA and differences among lesion subtypes (15). Conventional serum markers including CEA, CA19-9, CA242, and CA50 are widely used for surveillance and prognostic assessment but have limited value as standalone screening tests (16–19). Because fecal SDC2 methylation and serum markers reflect different biological processes, their combination may provide complementary diagnostic information. Nevertheless, evidence for this specific multimarker strategy remains limited.

Therefore, the present study aimed to evaluate the diagnostic performance of combining fecal SDC2 methylation with serum CEA, CA19-9, CA242, and CA50 for distinguishing CRC from non-CRC examination records in a retrospective hospital-based cohort. We collected paired fecal and serum measurements, evaluated individual and combined biomarker performance, analyzed correlations between fecal and serological markers, and assessed the incremental contribution of the serum markers. The study was exploratory and was not designed to establish population-screening performance.

## Materials and methods

### Patients

Patients diagnosed with CRC at the Department of Gastrointestinal Surgery, Wuhan the First People’s Hospital of Jiangxia District, Wuhan City, between July 2021 and November 2025 were consecutively enrolled. Inclusion criteria were as follows: pathologically confirmed CRC according to standard diagnostic guidelines, no prespecified age restriction was applied. Eligible examination records were required to have paired fecal SDC2 methylation and serum CEA, CA19-9, CA242, and CA50 measurements. Exclusion criteria included prior gastrointestinal surgery, any form of chemotherapy, radiotherapy, targeted therapy, or other antitumor treatments, a history of inflammatory bowel disease, or inadequate fecal sample quality. An age-matched cohort of patients diagnosed with colorectal adenoma during the same period served as the control group. In addition, we included a cohort of non-neoplastic individuals who visited our hospital during the same period. This retrospective study reviewed medical records of patients treated from 2021 to 2025. The study protocol, involving data collection and analysis from existing records, received ethical approval from the Ethics Committee of the First People’s Hospital of Jiangxia District, Wuhan City (2026K009). All data were retrieved and analyzed following approval, and patient confidentiality was strictly protected. All procedures were conducted in accordance with the principles of the Declaration of Helsinki, and informed consent was waived owing to the retrospective design.

### Fecal SDC2 methylation detection

Fecal SDC2 methylation was assessed using the Human SDC2 Gene Methylation Detection Kit (real-time fluorescence PCR method; Creative Biosciences [Guangzhou] Co., Ltd., Guangzhou, China) according to the manufacturer’s instructions (20). The commercial assay is an integrated workflow that includes stool DNA extraction, bisulfite conversion, and methylation-specific real-time PCR; therefore, no separate DNA extraction kit was used. Real-time PCR amplification was performed using a QuantStudio 5 Real-Time PCR System (Thermo Fisher Scientific, USA), with ACTB serving as the internal control gene. The PCR reaction volume was 30 μL, comprising 10 μL PCR reaction solution 1, 10 μL PCR reaction solution 2, and 10 μL template DNA. The amplification program consisted of an initial step at 95 °C for 5 min, followed by 48 cycles of 95 °C for 15 s, 58 °C for 30 s, and 72 °C for 30 s, with fluorescence detection in the FAM and Texas Red channels, followed by a final step at 40 °C for 30 s. Positive and negative controls were included in each analytical batch. Samples with an ACTB Ct value ≤ 36 were considered analytically valid. According to the manufacturer-defined criterion used in the multicenter clinical evaluation of this assay, an SDC2 Ct value < 38 was classified as methylation-positive, whereas Ct ≥38 or absence of detectable amplification was classified as negative (20). This binary cutoff was used only for assay interpretation; continuous SDC2 Ct values were used for quantitative comparisons and diagnostic modeling. The primer and probe sequences are proprietary components of the commercial assay and are not publicly disclosed by the manufacturer. No study-specific blinding procedure was implemented because the present study was a retrospective analysis of routinely generated clinical laboratory data.

### Serum tumor marker detection

Serum CEA, CA19-9, CA242, and CA50 concentrations were measured using commercially available magnetic microparticle chemiluminescence immunoassay kits (Shenzhen New Industries Biomedical Engineering Co., Ltd. [Snibe], Shenzhen, China) according to the manufacturer’s instructions. Venous blood samples (approximately 5 mL) were collected from each participant prior to colonoscopy or surgical intervention, and serum was separated by centrifugation at 3,000 rpm for 10 minutes. Normal reference values are as follows: CEA < 5.093 ng/mL, CA19-9 < 37.0 U/mL, CA242 < 20.0 U/mL, and CA50 levels < 25.0 U/mL. According to the analytical specifications of the commercial assays, the intra-assay and inter-assay coefficients of variation were ≤ 5% and ≤ 10%, respectively, for CEA; ≤ 8% and ≤ 10% for CA19-9; ≤ 8% and ≤ 10% for CA242; and ≤ 15% and ≤ 15% for CA50. All assays were performed according to the standardized procedures and quality-control requirements of the respective commercial kits.

### Statistical analysis

All statistical analyses were performed using GraphPad Prism 10 (GraphPad Software, San Diego, CA, USA) or Python 3.13.5. Continuous variables were expressed as median (interquartile range, IQR). Comparisons between groups were conducted using the Kruskal-Wallis test, followed by appropriate *post-hoc* pairwise comparisons where applicable. Correlations between SDC2 Ct values and serum tumor markers were assessed using Spearman’s rank correlation coefficient. Multiple testing in correlation analyses was adjusted using the Benjamini-Hochberg false discovery rate (FDR) correction. Multivariable logistic regression was used to construct the combined diagnostic models, with CRC status as the binary outcome and fecal SDC2 Ct value and serum CEA, CA19-9, CA242, and CA50 concentrations entered as continuous predictors. The logistic regression coefficients of the four diagnostic models were shown in **Supplementary Table S1**. Four prespecified nested models were evaluated: SDC2 alone (Model 1); SDC2 plus CEA (Model 2); SDC2 plus CEA and CA19-9 (Model 3); and SDC2 plus CEA, CA19-9, CA242, and CA50 (Model 4). For the full model, the fitted logistic regression equation was: logit(P) = 19.775 - 0.691 × SDC2 Ct + 0.552 × CEA + 0.0535 × CA19-9 + 0.0111 × CA242 + 0.0312 × CA50, where P denotes the predicted probability of CRC. The corresponding probability was calculated as P = exp(logit(P))/[1 + exp(logit(P))]. No data-driven variable-selection procedure was applied. Predicted probabilities generated from the logistic regression models were used for ROC analysis. AUCs were reported with 95% confidence intervals (CI), and correlated ROC curves were compared using DeLong’s test. Bonferroni correction was applied for multiple AUC comparisons across models. The optimal operating threshold was determined using the Youden index. Sensitivity, specificity, positive predictive value, and negative predictive value were calculated with 95% CI. Internal validation was performed using patient-grouped 10-fold cross-validation and patient-cluster bootstrap resampling. Model calibration was evaluated using calibration plots, calibration slope, and the Brier score. Incremental predictive performance was further assessed using the integrated discrimination improvement (IDI), continuous net reclassification improvement (NRI), and decision curve analysis (DCA) to quantify clinical net benefit. To evaluate the potential influence of available demographic confounders, a sensitivity model additionally adjusted the full biomarker model for age and sex. Because detailed lesion-grade and comorbidity information was not systematically available, these variables could not be incorporated into the adjustment model. Additional sensitivity analyses evaluated diagnostic performance separately for CRC versus adenoma/polyp and CRC versus non-tumor/inflammatory examination records. A P value < 0.05 was considered statistically significant. For multiple comparisons, corrected P values (FDR or Bonferroni) were used for significance determination.

## Results

### Levels of fecal SDC2 methylation and serum tumor markers in patients

A total of 354 examination records from 325 individuals were included in the primary analysis, comprising 55 CRC, 188 adenoma/polyp, 106 non-tumor/inflammatory, and 5 other/unclear examination records (**Figure 1**). The median age was 60 years (IQR, 48.0-69.8), and 271 records (76.6%) were from male patients. Baseline demographic and biomarker characteristics are summarized in **Table 1**. The adenoma/polyp group was analyzed as an aggregate category because detailed lesion-level endoscopic and histopathological characteristics required for standardized classification of advanced adenomas were not systematically available for all examination records. Fecal methylation analysis revealed that SDC2 methylation levels differed significantly among the four groups (**Figure 2**). Compared with patients with adenomas or other non-tumor lesions, serum levels of CEA, CA19-9, and CA50 were relatively higher in patients with CRC (**Table 1**). Spearman correlation analysis showed that the Ct values of SDC2 methylation exhibited a weak negative correlation with serum levels of CEA (r = -0.1331, 95% CI, -0.2370--0.0262; P = 0.0122) and CA19-9 (r = -0.1119, 95% CI, -0.2174--0.004; P = 0.0366), but no significant correlation with CA242 (r = -0.0463, 95% CI, -0.1533-0.0617; P = 0.3867) or CA50 (r = -0.0371, 95% CI, -0.1440-0.0706; P = 0.4872) (**Figure 3**).

**Figure 1:**
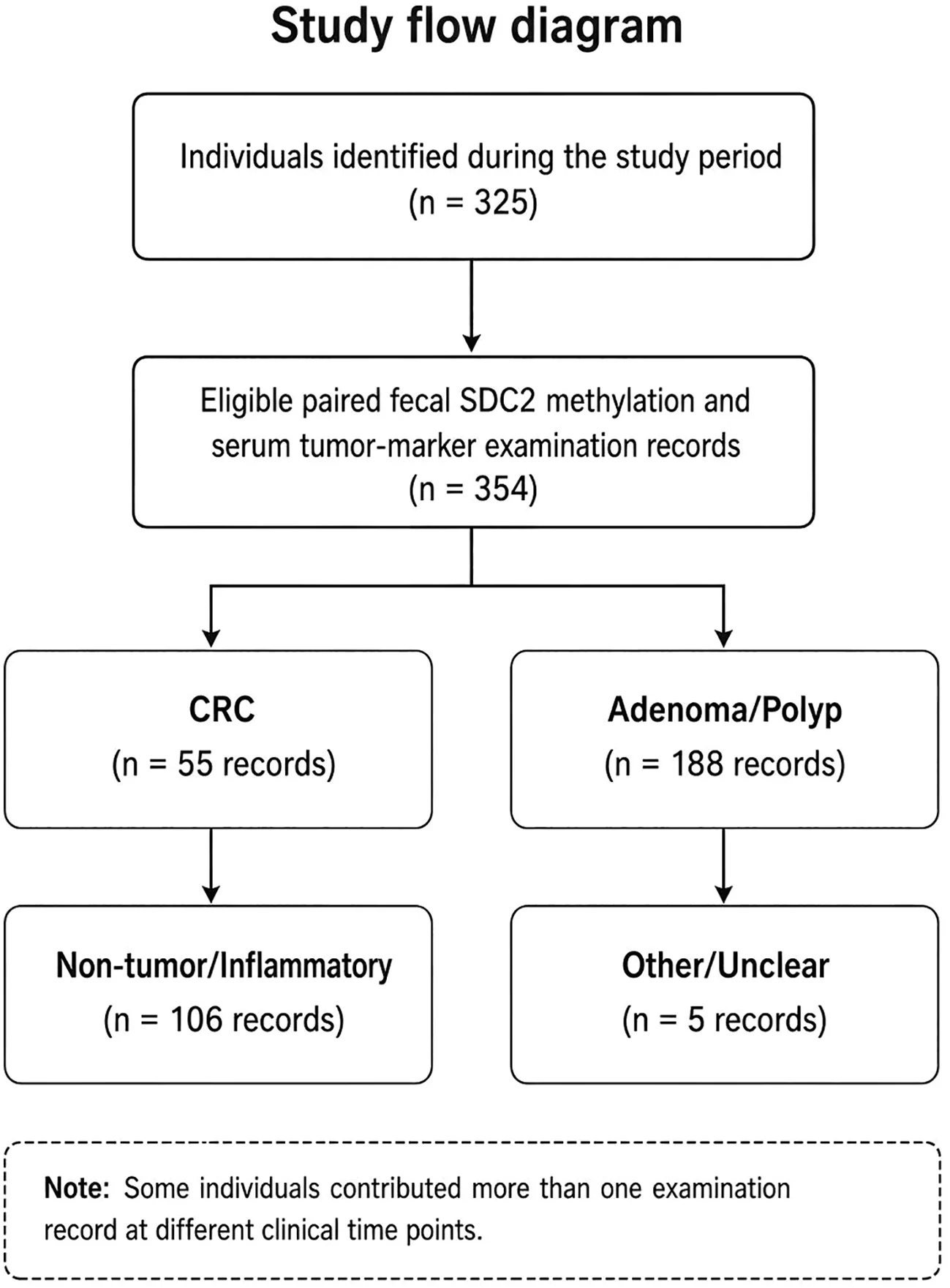
Flowchart of sample inclusion. A total of 354 examination records from 325 individuals were included in the final analysis, consisting of 55 CRC records, 188 adenoma/polyp records, 106 non-tumor/inflammatory records, and 5 other/unclear records.

**Table 1:** Baseline demographic and biomarker characteristics of the included examination records .

Characteristics	Overall (n = 354)	CRC (n = 55)	Adenoma/polyp (n = 188)	Non-tumor/inflam (n=106)	Other/unclear (n=5)
Age, years median (IQR)	60.0 (48.0-69.8)	60.0 (49.0-69.0)	61.0 (49.0-69.3)	58.0 (46.0-68.8)	75.0 (63.0-77.0)
Male, n (%)	271 (76.6)	48 (87.3)	138 (73.4)	82 (77.4)	3 (60.0)
Female, n (%)	83 (23.4)	7 (12.7)	50 (26.6)	24 (22.6)	2 (40.0)
SDC2, median (IQR)	–	34.95 (33.25-35.91)	36.76 (35.95-37.44)	36.98 (36.18-37.54)	35.77 (35.77-37.26)
CEA, median (IQR)	–	3.59 (2.56-5.36)	2.58 (1.42-3.68)	2.44 (1.42-3.61)	2.26 (0.98-3.75)
CA19, median (IQR)	–	23.13 (15.11-35.06)	16.83 (9.92-23.77)	17.58 (10.82-24.84)	12.50 (10.45-28.93)
CA242, median (IQR)	–	8.40 (6.05-13.07)	9.03 (5.10-13.80)	10.12 (4.38-13.25)	8.96 (5.23-14.45)
CA50, median (IQR)	–	13.54 (8.54-18.51)	12.06 (5.95-17.68)	11.62 (6.79-15.95)	12.57 (6.70-12.59)

Data are presented at the examination-record level. A total of 354 examination records were obtained from 325 individuals; some individuals contributed examinations at different clinical time points.

**Figure 2:**
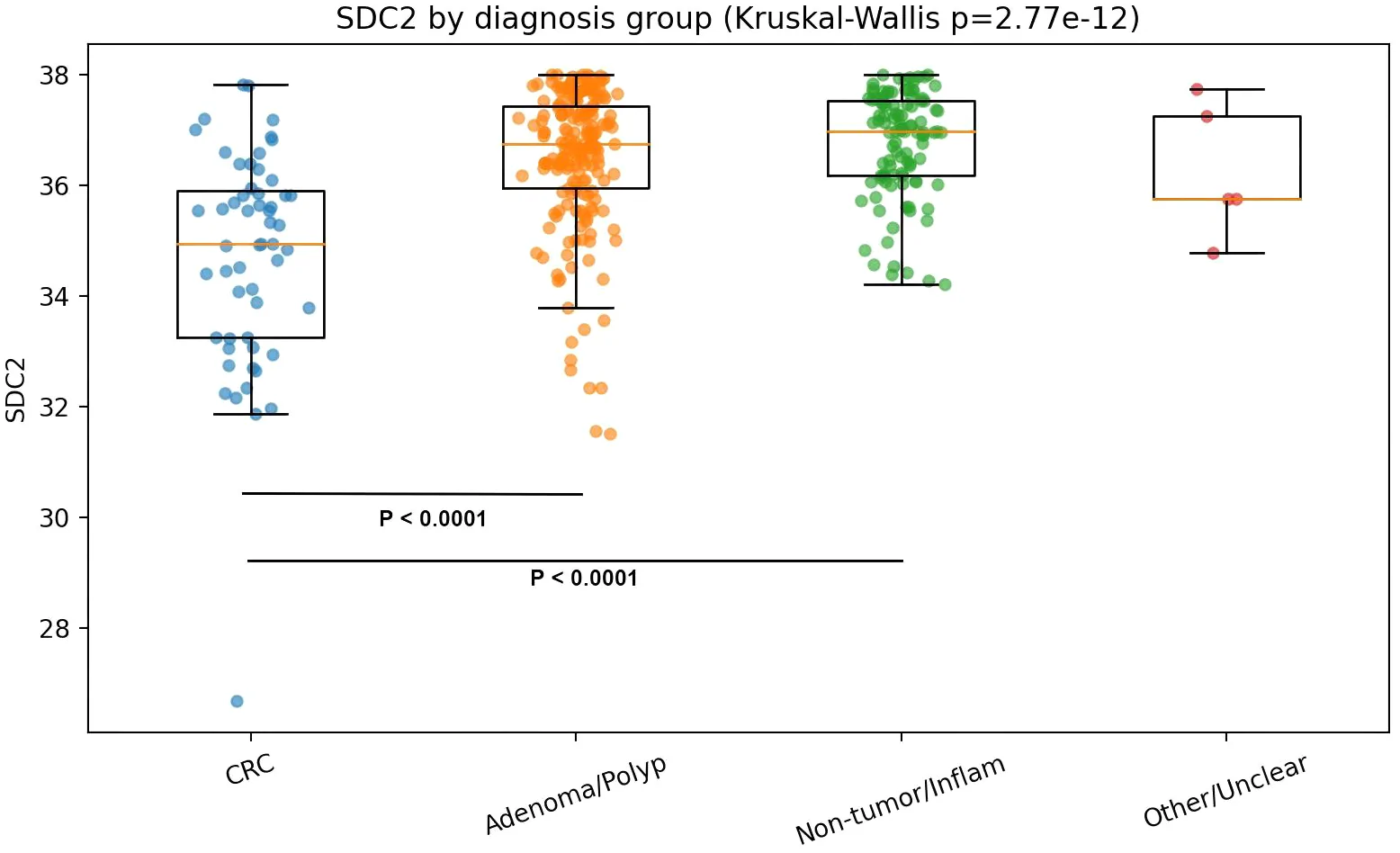
Comparison of fecal SDC2 methylation levels among different gastrointestinal lesion groups. A total of 354 examination records from 325 enrolled participants were divided into four groups: colorectal cancer (CRC, n=55), adenoma/polyp (n=188), non-tumor/inflammatory lesion (n=106), and other/unclear lesions (n=5). Fecal methylation detection verified that significant differences were observed for CRC versus adenoma/polyp (adjusted P < 0.0001) and CRC versus non-tumor/inflammatory conditions (adjusted P < 0.0001).

**Figure 3:**
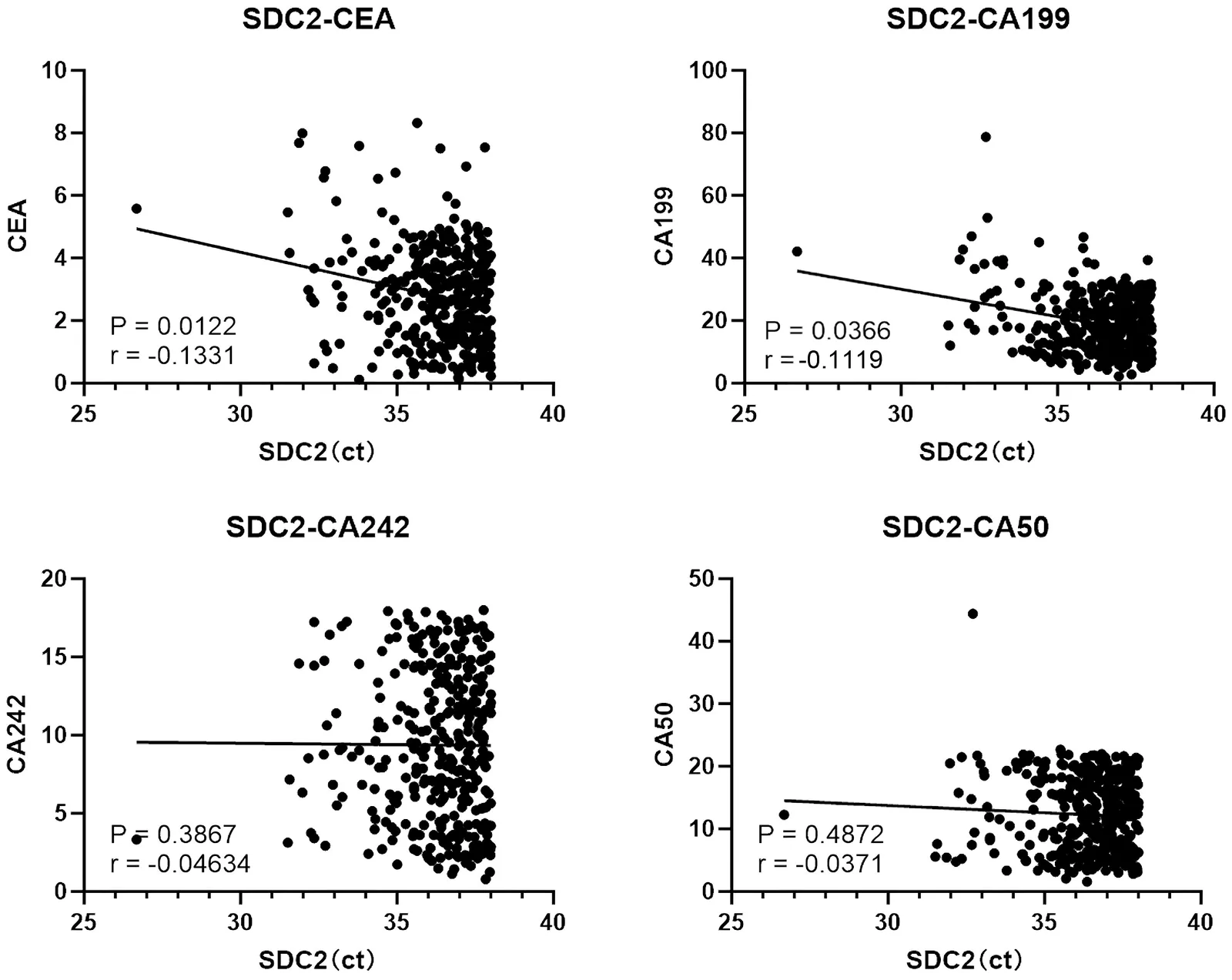
Spearman correlation analysis between fecal SDC2 methylation Ct values and serum tumor marker levels. The Ct values of fecal SDC2 methylation showed significant weak negative correlations with serum CEA (r = -0.1331, P = 0.0122) and CA19-9 (r = -0.1119, P = 0.0366). No statistically significant correlation was observed between SDC2 methylation Ct values and serum CA242 (P = 0.3867) or CA50 (P = 0.4872).

### Evaluation of the clinical diagnostic value of fecal SDC2 methylation, serum tumor markers, and their combination

To evaluate the clinical diagnostic value of these markers in distinguishing CRC from non-tumor controls, ROC curve analysis was performed. The AUC values for SDC2 methylation, CEA, CA19-9, CA242, and CA50 were 0.814, 0.709, 0.666, 0.495, and 0.567, respectively (**Figure 4**), indicating that CA19-9, CA242, and CA50 alone had very limited clinical diagnostic value for CRC. Further multivariable logistic regression was used to integrate fecal SDC2 Ct values with serum tumor markers. The AUC was 0.814 (95% CI, 0.751-0.876) for SDC2 alone and increased to 0.866 (95% CI, 0.814-0.918) after addition of CEA. Further inclusion of CA19–9 resulted in an AUC of 0.872 (95% CI, 0.820-0.925), whereas the full model incorporating CEA, CA19-9, CA242, and CA50 yielded an AUC of 0.872 (95% CI, 0.818-0.926) (**Figure 5**). Although CA242 and CA50 were included in the prespecified full model, their additional contribution to discrimination was limited. The AUC was 0.872 for the model incorporating SDC2, CEA, and CA19–9 and remained essentially unchanged at 0.872 after further addition of CA242 and CA50. In the full multivariable model, CA242 and CA50 were not independently associated with CRC status (P = 0.784 and P = 0.335, respectively) (**Supplementary Table S1**). In a sensitivity analysis additionally adjusting the full biomarker model for age and sex, diagnostic discrimination was essentially unchanged (AUC, 0.879 versus 0.872 for the unadjusted biomarker model; **Supplementary Table S2**), suggesting that the principal findings were not materially driven by these available demographic variables. These findings suggest that integration of continuous SDC2 Ct values with serum tumor markers provides modestly improved discrimination of CRC from non-CRC examination records.

**Figure 4:**
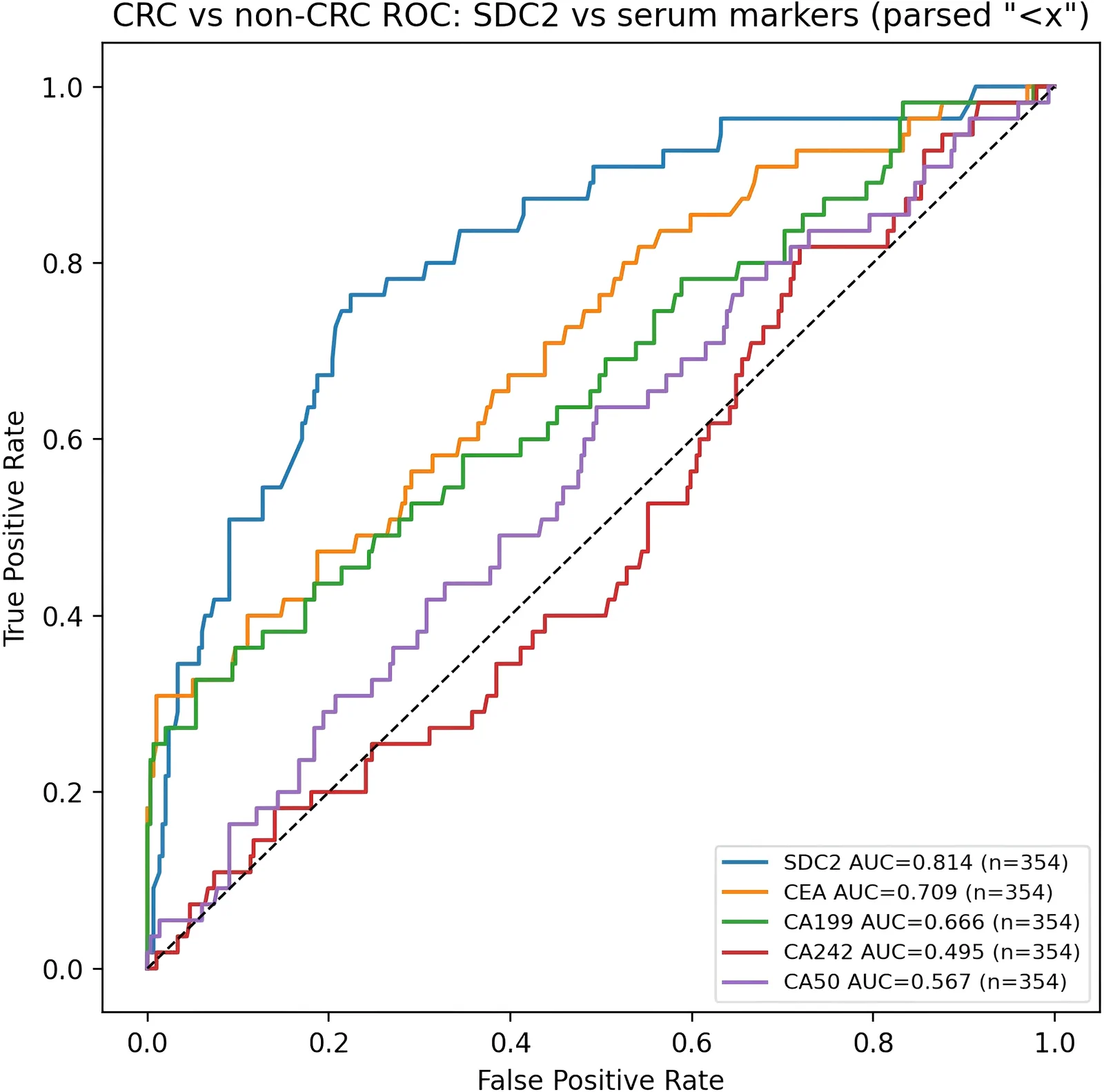
ROC curve analysis for the single diagnostic performance of fecal SDC2 methylation and four serum tumor markers (CEA, CA19-9, CA242, CA50) in distinguishing CRC from non-tumor gastrointestinal lesions. The corresponding AUC values of single indicators were 0.814 (SDC2 methylation), 0.709 (CEA), 0.666 (CA19-9), 0.495 (CA242), and 0.567 (CA50), indicating limited independent diagnostic value of CA19-9, CA242 and CA50 for CRC detection.

**Figure 5:**
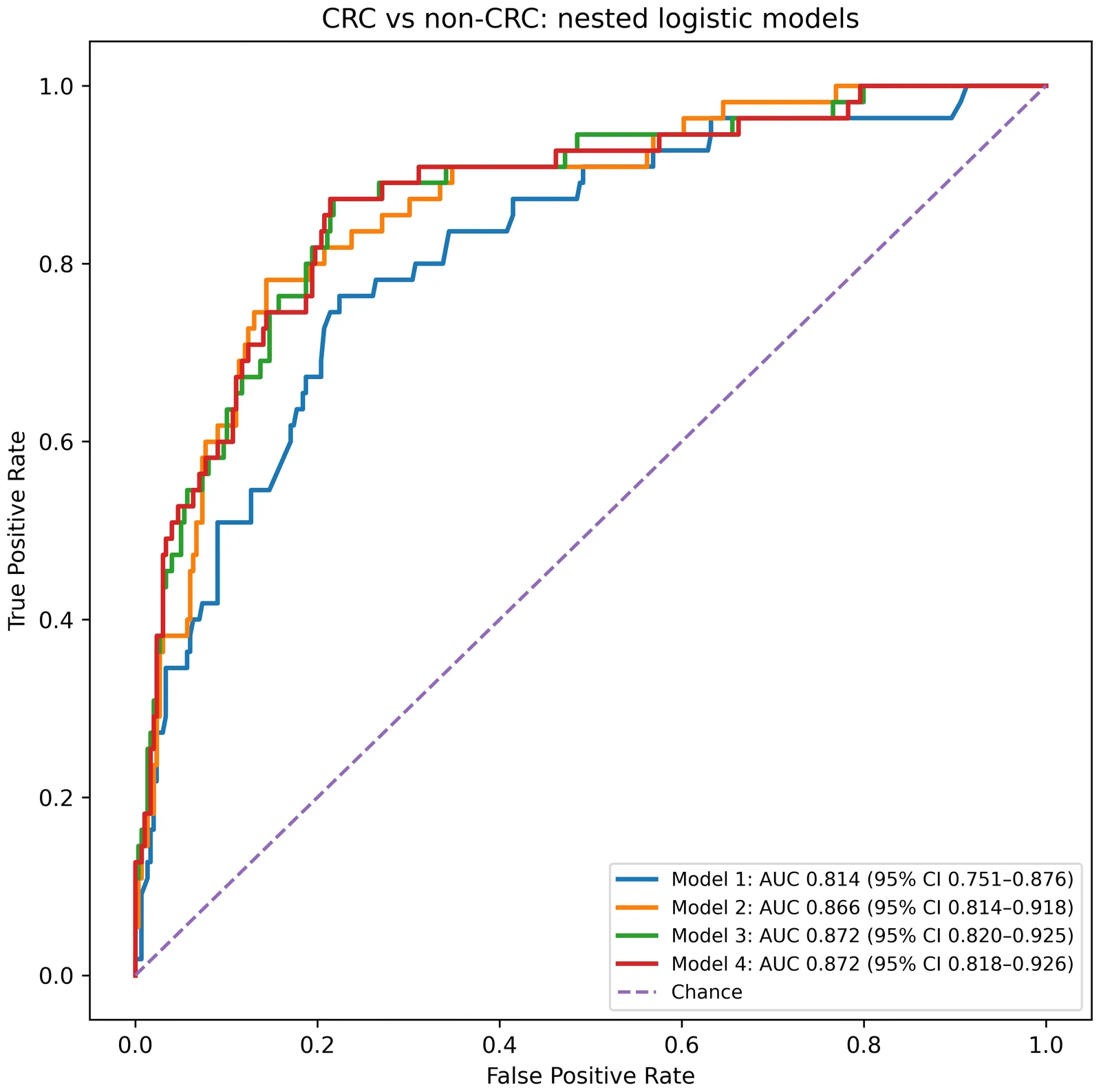
ROC curve analysis of combined diagnostic models integrating fecal SDC2 methylation and serum tumor markers for CRC identification. The AUC value was 0.814 (95% CI: 0.751-0.876) for SDC2 methylation alone. The AUC increased to 0.866 (95% CI: 0.814-0.918) after combination with CEA, and further rose slightly to 0.872 (95% CI: 0.818-0.926) when incorporating all four serum markers (CEA, CA19-9, CA242, CA50). SDC2 alone (Model 1); SDC2 plus CEA (Model 2); SDC2 plus CEA and CA19-9 (Model 3); and SDC2 plus CEA, CA19-9, CA242, and CA50 (Model 4).

### Additional evaluation of the combined diagnostic models

Compared with SDC2 alone, the increase in AUC for the full combined model was modest and did not reach conventional statistical significance by DeLong’s test (ΔAUC = 0.058, P = 0.055). The full model included five biomarker predictors and 55 CRC examination records, corresponding to approximately 11 CRC events per predictor. The estimated *post-hoc* power for detecting the observed AUC increment of approximately 0.058 was 48%, indicating limited statistical power for this modest incremental effect. At the Youden-optimal threshold, the full model achieved a sensitivity of 87.3% (95% CI, 76.0%-93.7%), specificity of 78.6% (95% CI, 73.6%-82.9%), PPV of 42.9% (95% CI, 34.1%-52.1%), and NPV of 97.1% (95% CI, 94.2%-98.6%) (**Supplementary Table S3**).

Internal validation yielded a patient-grouped 10-fold cross-validated AUC of 0.854 and a bootstrap optimism-corrected AUC of approximately 0.858 for the full model. The cross-validated Brier score was 0.095, with a calibration slope of 0.864. Compared with SDC2 alone, the full model yielded an IDI of 0.098 (95% CI, 0.043-0.154; P = 0.0004) and a continuous NRI of 0.657 (95% CI, 0.360-0.941; P < 0.001) (**Supplementary Table S4**; **Supplementary Figure S1A**). Decision curve analysis showed a generally higher net benefit for the combined model than for SDC2 alone across the examined threshold probabilities (**Supplementary Figure S1B**).

## Discussion

Early detection remains central to reducing CRC mortality (4, 21). Colonoscopy is effective but invasive and resource-intensive, whereas FIT has limited sensitivity for premalignant lesions (5, 6). Against this background, the present study evaluated whether routinely available serum tumor markers provide incremental diagnostic information when added to fecal SDC2 methylation. Because the cohort was retrospective and hospital-based and lacked complete stage-specific data, the findings should be interpreted as evidence of CRC discrimination rather than as direct validation of a population-screening or early-stage detection strategy.

SDC2 belongs to the SDC family of type I transmembrane heparan sulfate proteoglycans, with the ability to bind laminin. It is synthesized by tumor cells and contributes significantly to tumor angiogenesis, invasion, and metastasis by activating tumor-derived fibroblasts and associated signaling pathways (22). Notably, the methylation level of SDC2 is elevated in tumor tissues compared to adjacent non-tumor tissues, establishing it as a promising methylated biomarker for CRC. Consistent with previous findings (23), our results demonstrated that the Ct value of fecal SDC2 methylation was significantly lower in CRC samples compared to those from patients with adenomas/polyps, non-tumor/inflammatory lesions, and other undetermined conditions, indicating that SDC2 methylation levels are relatively higher in CRC. ROC analysis showed that the AUC for SDC2 was 0.814, which is comparable to the findings reported by Long et al. (23) and considerably higher than that of the four serum tumor markers. Recent multicenter prospective and case-control studies have further validated the stable diagnostic value of stool SDC2 methylation for CRC and advanced colorectal neoplasia in Chinese and asymptomatic high-risk populations (13, 14), supporting the reliability of our core findings.

The clinical application of fecal SDC2 testing is limited by the heterogeneous distribution of methylated DNA in stool samples and its reduced sensitivity for certain lesion subtypes (15). Accordingly, combining SDC2 with additional methylation markers or serum proteins has been explored to enhance the specificity and sensitivity of early CRC diagnosis (24, 25). Mechanistically, fecal SDC2 methylation and serum tumor markers reflect distinct yet complementary biological processes. Fecal SDC2 methylation originates from methylated DNA shed by exfoliated tumor epithelial cells into the colonic lumen; its detection is therefore spatially confined to the local tumor site, which may explain its variable sensitivity depending on tumor location, size, and shedding efficiency (26), In contrast, serum markers including CEA, CA19-9, CA242, and CA50 capture systemic host-tumor interactions, such as tumor burden, inflammation, immune response, and aberrant glycosylation within the tumor microenvironment (27, 28). This complementary biological basis, local DNA shedding versus systemic protein alterations, provides a rationale for combining these biomarkers to enhance diagnostic performance across a broader spectrum of CRC presentations (29). CEA is a routinely used serum marker in CRC surveillance, primarily applied for postoperative recurrence monitoring and assessment of treatment efficacy (30, 31). CA19-9, a sialylated Lewis antigen expressed in pancreatic, biliary and gastrointestinal epithelial cells, serves as a standard serum biomarker for pancreatic and biliary tract malignancies (32, 33); in CRC, it acts as a complementary marker to CEA, particularly for CEA-negative tumors or mucinous subtypes (34). CA242 is a sialylated sphingolipid antigen expressed on malignant gastrointestinal cells, and its serum levels correlates with T and N stages, perineural invasion, and pTNM stage, suggesting its prognostic value in CRC (19, 35). CA50 is a broad-spectrum high-molecular-weight glycoprotein antigen detected in various malignant tumors, including pancreatic, cervical, and colorectal cancers (36, 37), and its aberrant expression links to metastasis in CRC (38). Our combined panel of fecal SDC2 with the four serum markers increased the AUC from 0.814 (SDC2 alone) to 0.872. This performance outperformed single fecal occult blood test (FOBT) (AUC = 0.7532) (24), SDC2 combined with NDRG4 methylation (AUC = 0.850) (23), and the four-marker serum panel alone (AUC = 0.80) (39). Moreover, diagnostic accuracy of our panel was equivalent to FOBT combined with these four serum markers for early CRC diagnosis (AUC = 0.87) (39). Although our panel was inferior to the triple panel integrating FOBT, CEA and multiple methylation markers (SDC2/SFRP2/TFPI2, AUC = 0.9725) (24), our approach offers favorable clinical accessibility. Notably, the incremental contribution of the individual serum markers was heterogeneous. CEA and CA19–9 accounted for most of the improvement in discrimination, whereas further addition of CA242 and CA50 produced essentially no additional increase in AUC. This finding is consistent with the poor standalone diagnostic performance of CA242 and CA50 in the present cohort and suggests that increasing the number of serum markers does not necessarily translate into clinically meaningful improvement.

Although integration of the four serum tumor markers increased the AUC from 0.814 for SDC2 alone to 0.872 for the full model, the absolute improvement in discrimination was modest, and the DeLong comparison did not reach the conventional threshold for statistical significance. Therefore, the incremental value of the combined panel should not be interpreted solely on the basis of AUC. Supporting analyses, including IDI, NRI, and decision curve analysis, suggested improved risk discrimination and net benefit compared with SDC2 alone. Internal validation using patient-grouped cross-validation and cluster bootstrap resampling further demonstrated that the diagnostic performance was largely retained after correction for optimism. Nevertheless, these findings were derived from a retrospective single-center cohort, and external prospective validation remains necessary before clinical implementation.

Established stool-based molecular assays provide important context for interpreting the present findings. In the pivotal study of first-generation multitarget stool DNA testing, which combined molecular stool markers with fecal hemoglobin, sensitivity for CRC was 92.3% versus 73.8% for FIT, while sensitivity for advanced precancerous lesions was 42.4% versus 23.8% (6). Next-generation multitarget stool DNA testing has maintained high CRC sensitivity while improving specificity (10). ColoClear, which incorporates KRAS mutation, NDRG4 and BMP3 methylation, and fecal hemoglobin, has also shown favorable diagnostic performance in Chinese populations (11). SDC2-based assays such as EarlyTect provide a more focused methylation-based approach (12). These platforms differ in biomarker composition, positivity algorithms, intended-use populations, and regulatory context. Therefore, direct numerical comparison with the present retrospective analysis is inappropriate. The AUC of 0.872 observed here should be interpreted only as evidence that serum markers may add complementary information to a stool SDC2 signal within this cohort, not as evidence of superiority or equivalence to established commercial screening assays.

From a translational and implementation perspective, the clinical adoption of our combined five-marker panel requires careful consideration of cost-effectiveness, laboratory feasibility, and workflow integration. Adding four serum markers inevitably increases assay cost, laboratory workload, and panel complexity compared to fecal SDC2 testing alone, and the clinical necessity of routinely including CA242 and CA50 remains uncertain. Nevertheless, these serum markers are measured using standard automated immunoassay platforms widely available in most clinical laboratories, requiring no additional equipment or specialized training, which facilitates seamless workflow integration. Moreover, all four markers can be measured simultaneously from a single blood draw, minimizing additional patient visits and processing time. From a screening perspective, blood-based testing offers higher patient acceptance and compliance than stool collection, with objective quantitative readouts reducing interpretation variability. However, because patient-level cost, reimbursement, downstream diagnostic utilization, and health-outcome data were not available in this retrospective study, a formal cost-effectiveness analysis could not be performed. Therefore, although the full five-marker panel was retained as the prespecified model evaluated in this study, formal health economic analyses, including incremental cost-effectiveness ratios, are needed to determine whether the diagnostic gain justifies the additional costs before this strategy can be recommended for population-based screening.

There are several limitations of this study. First, this was a retrospective, single-center study restricted to a single geographic region and a single ethnicity, and no independent external validation cohort was available. Internal cross-validation and bootstrap correction can estimate optimism but cannot replace validation in a genuinely independent population. Second, the prevalence of CRC in our hospital-based cohort (approximately 17%) was substantially higher than that expected in a general screening population, which may inflate PPV and limits direct generalization to community screening. Third, the relatively small number of CRC cases (n = 55) limited statistical power and increased the possibility of overfitting; the *post-hoc* power for the observed AUC increment was only 48%. Fourth, complete TNM staging information was not systematically available, preventing stage-specific evaluation, particularly for stage I-II disease. Accordingly, the present data cannot establish early-stage sensitivity. Fifth, tumor location, histological subtype, smoking history, family history, and other clinical covariates were not systematically available, so **Table 1** cannot provide the requested comprehensive clinicopathological characterization and residual confounding cannot be excluded. Sixth, the adenoma/polyp group lacked standardized lesion-level endoscopic and histopathological classification, preventing separate evaluation of advanced adenomas, non-advanced adenomas, and sessile serrated lesions. Accordingly, the present study cannot establish performance for advanced precancerous lesions. These limitations mean that the model should be regarded as exploratory. Prospective multicenter studies with prespecified sample-size planning, independent validation, stage-specific analyses, comprehensive clinicopathological characterization, advanced-precancerous-lesion endpoints, and health-economic evaluation are required before clinical screening implementation.

The broader CRC precision-biomarker landscape now extends beyond stool methylation and conventional serum markers to ctDNA, genomic and immune biomarkers, microbiome signatures, radiomics, patient-derived organoids, and integrated multi-omics approaches (40–44). These modalities address different clinical questions, ctDNA has particular relevance to minimal residual disease and treatment monitoring, genomic and immune biomarkers can guide molecularly targeted or immunotherapeutic strategies, and organoid/multi-omics approaches are being explored for individualized treatment selection and biological stratification. In this context, the present stool-plus-serum approach should be viewed as complementary rather than competitive. Its potential role, if prospectively validated, would be as a comparatively accessible front-end diagnostic strategy, whereas more complex precision platforms may be better suited to downstream risk stratification, treatment selection, and disease monitoring.

In conclusion, combining fecal SDC2 Ct values with serum tumor markers produced a modest improvement in CRC discrimination in this retrospective hospital-based cohort. Most of the incremental information was contributed by CEA and CA19-9, whereas CA242 and CA50 added little further discrimination. These findings do not establish population-screening effectiveness or early-stage diagnostic performance. Independent prospective validation, including stage-specific and advanced-precancerous-lesion assessment, is required before the combined model can be considered for clinical screening use.

## Data Availability

The original contributions presented in the study are included in the article/**Supplementary Material**. Further inquiries can be directed to the corresponding authors.
